# Hospitalization Events Among Adolescents and Adults With Sickle Cell Disease in a Tertiary Care Center in Central India

**DOI:** 10.7759/cureus.61185

**Published:** 2024-05-27

**Authors:** Preetam Wasnik, Pranita Das, Ajit Kumar, Pankaj K Kannauje, Rohini R, Vinay Pandit, Tarun Sahu, Jyoti Sahu

**Affiliations:** 1 General Medicine, All India Institute of Medical Sciences, Raipur, Raipur, IND; 2 General Medicine, Shri Balaji Institute of Medical Science, Raipur, IND; 3 Medicine, All India Institute of Medical Sciences, Raipur, Raipur, IND

**Keywords:** hospitalization, infection, acute chest syndrome, painful crisis, sickle cell disease

## Abstract

Background: Sickle cell disease (SCD) is an inherited red blood cell disorder, wherein mutation causes the substitution of glutamic acid to valine at the sixth position of the β-globin chain. These include sickle cell anemia (homozygous sickle mutation), sickle-beta thalassemia, and hemoglobin SCD. The clinical manifestations of SCD are protean. Individuals with SCD suffer from both acute and chronic complications, which include recurring episodes of pain commonly called vaso-occlusive crisis (VOC) - acute chest syndrome (ACS); aseptic necrosis of the bone; micro-infarction of the spleen, brain, and kidney; infections; stroke; and organ damage affecting every part of the body. SCD necessitates frequent hospitalizations because of severe complications, which pose a significant burden on caregivers and economic strain on healthcare systems. The pattern of hospital admission with SCD varies in different parts of the world.

Objective: This study aimed to determine the causes of hospitalization among adolescent and adult patients with SCD and to determine factors associated with their hospital stay.

Methods: The study was a hospital-based prospective observational study comprising adolescent and adult patients diagnosed with SCD, aged 15-45 years, who were hospitalized in the Department of General Medicine at All India Institute of Medical Sciences in Raipur from August 2021 to August 2022.

Result: According to our study, the primary reason for hospitalization was a painful crisis, accounting for 63% of cases, followed by infection (17%), ACS (11%), and acute hemolytic crisis (9%). Notably, we did not observe any significant differences between genders and causes of admission (p > 0.05). Joint pain (p = 0.005), back pain (p = 0.001), and chest pain (p = 0.001) were more frequently reported by adults over the age of 19. In addition, our analysis of the duration of hospital stays and various factors revealed that patients admitted for infections had a significantly longer mean hospital stay duration (p = 0.040).

Conclusion: Acute painful crises were the primary cause of hospital admission among individuals with SCD; many patients also encountered infections and ACS. Furthermore, patients who experienced infections and VOC had a lengthier duration of hospital stay. Therefore, it is essential to provide them with comprehensive instructions on various preventive measures against infections and the factors that trigger painful crises.

## Introduction

Sickle cell disease (SCD) and its variants are a type of hemoglobinopathy arising from a genetic mutation that replaces glutamic acid with valine at the sixth position of the β-globin chain of hemoglobin [1,2]. This genetic disorder is prevalent in numerous regions, including sub-Saharan Africa, the Mediterranean Basin, the Middle East, and regions in India. SCD usually manifests early in childhood, the most common being vaso-occlusive crisis, followed by bone pain, anemia, infection, splenic sequestration, and aplastic crisis [3]. Its substantial morbidity and mortality make it a significant public health concern [4]. Although the mutation in individuals with SCD remains identical, the clinical manifestations vary significantly, encompassing from a benign, almost asymptomatic presentation to a severe and potentially life-threatening condition [5-7]. Individuals with SCD often experience chronic anemia due to premature hemolysis caused by the inflexible nature of sickled cells, which are severely limited in their capacity to adjust to their surroundings, particularly within the microvasculature. Sickling is triggered by fever, dehydration, hypoxia, acidosis, stress, and exposure to cold temperatures. However, in many cases, a precursor event leads to sickling that cannot be explicitly identified.

Given our current understanding of the multifaceted clinical presentations of sickle cell hemoglobinopathies, a typical individual with SCD experiencing a crisis may exhibit a range of clinical and laboratory indicators. For instance, episodes of painful crises frequently involve ischemia, pain, tenderness, fever, hand and foot syndrome, priapism, and chronic leg ulcers accompanied by secondary infections. Acute chest syndrome may manifest as chest pain, tachypnea, fever, cough, and diminished oxygen saturation. Moreover, SCD commonly leads to widespread renal papillary necrosis, bone and joint ischemia, and avascular necrosis of the femur. SCD patients also experience chronic hemolytic anemia and are vulnerable to sudden and severe life-threatening complications. The acute sickling of red blood cells (RBCs) may cause pain, impairment of organs, and permanent damage from recurring sickling events [8-11]. Severe SCD complications often necessitate hospitalization, posing a significant challenge for caregivers. The hospitalization patterns for SCD patients vary globally, with acute painful crises serving as the primary reason for admissions in certain regions, and infections remain the leading cause of hospitalizations in developing countries [12-15]. Despite a rate of 0.8 painful crises per year, many individuals with SCD receive inadequate treatment in emergency departments [11]. SCD patients necessitate hospitalization for disease-related complications, and a considerable number require readmission. Hence, identifying factors that can predict the likelihood and duration of hospitalization can alleviate the disease burden and enhance the quality of life.

Several studies have examined the clinical manifestations of SCD, including variations in hemoglobin variants that are influenced by genetic, ethnic, and regional differences [16-21]. Identifying the factors that predict hospitalizations and duration of hospital stay related to SCD could result in better health outcomes, quality of life, and reduced morbidity. The present study aimed to investigate the reasons for hospitalization among adolescent and adult SCD patients in central India (Raipur) and identify the factors linked to the duration of hospital stays.

## Materials and methods

This was a hospital-based prospective observational study conducted at All India Institute of Medical Sciences (AIIMS), Raipur, C.G., India. This study included 100 adolescents and adult SCD patients aged 15-45 years hospitalized for various reasons in the General Medicine Ward of All India Institute of Medical Sciences, Raipur, between August 2021 and August 2022.

Individuals aged 15-45 years and confirmed SCD patients who gave consent were included in the study. Pregnant patients and individuals below 15 years and above 45 years were excluded. A total of 100 SCD patients were selected as the study population and the sample size was taken based on the convenience of the study. A detailed history and clinical examination of enrolled patients was done according to the pre-structured proforma. Blood samples were collected for laboratory parameters. Morbid events such as an acute painful crisis, severe anemia, sequestration, acute febrile illness, and stroke were defined as per standard definitions. The hospitalization event, including the cause of hospitalization, was noted. A comprehensive clinical data sheet was created in which information such as the age at presentation, sex, and previous hospitalization history was noted. Furthermore, a thorough clinical examination was conducted for each patient, which included a general checkup, vital signs measurements, and systemic examination. The number of hospitalizations and blood transfusions in the previous year was recorded. Disease severity was defined as ≥3 admissions and/or ≥3 blood transfusions in the previous year; treatments received, the duration of hospitalization, complications, and patient outcomes were also documented. Data entry was done in Excel, and analysis was done by Statistical Product and Service Solutions (SPSS, version 20; IBM SPSS Statistics for Windows, Armonk, NY) software.

It is noteworthy to state that all facets related to the management of patients at AIIMS Raipur are offered free of charge. Before enrollment in the study, informed consent had been taken from patients or their parents. Those who did not provide valid written consent were excluded from the study. This research was carried out following the ethical principles of the World Medical Association Code of Ethics (Declaration of Helsinki) for studies involving human subjects. The study was commenced after approval from the Institute Ethical Committee at AIIMS Raipur (Proposal No. AIIMSRPR/IEC/2020/579).

Statistics

The statistical analysis was conducted using the SPSS program. Categorical variables are exhibited in the form of percentages and proportions. The assessment of statistical significance was made using Pearson's chi-square test for categorical variables. The quantitative data were represented as mean ± standard deviation (SD), and the comparison of differences between the two means was accomplished using 𝑡-tests. Statistical tests with P < 0.05 were considered significant.

## Results

A total of 100 adolescent and adult patients with SCD were seen in the hospital in this study period. Of these 100 patients, 59 were males, and 41 were females. Patients were divided into two age groups: 25 patients were from >15-19 years old, and 75 patients were >19 years old. Regarding their residence, 41 patients are from urban, and 59 patients are from rural areas (Table 1).

**Table 1:** Basic demographic characteristics of the study population

Basic Characteristics	Study Group (n=100)
	Number
Age	
>15-19	25
>19	75
Sex	
Male	59
Female	41
Residence	
Urban	41
Rural	59
Duration of hospitalization (Mean ± SD)	10.79 ± 7.01
Hematological Parameters (Mean ± SD)	
Hemoglobin	8.05 ± 1.85
MCV	84.34 ± 13.97
MCH	27.74 ± 4.73
MCHC	32.42 ± 2.12
RDW	19.7 ± 3.56
WBC	11.8 ± 8.02
Platelets	246.33 ± 191.85

**Figure 1:**
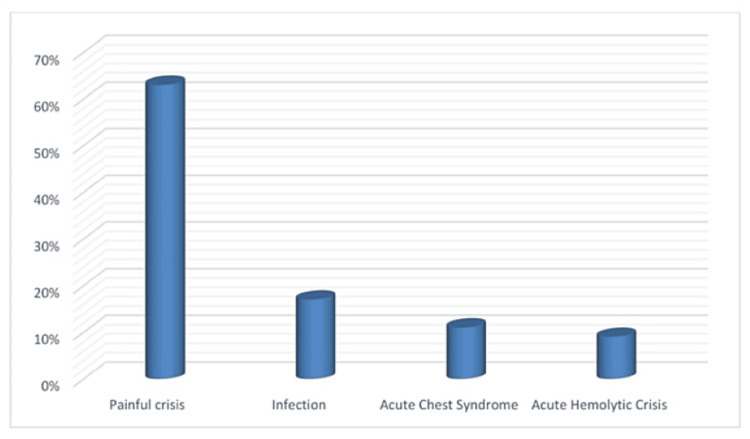
Bar chart showing different causes of hospitalization among SCD patients SCD, Sickle cell disease

The reasons for hospitalization among SCD patients are depicted in Figure 1. In the present investigation, the leading reason for hospitalizations in individuals with SCD was identified as painful crises (vaso-occlusive crisis, VOC) (63%), and infections constitute the second leading reason for hospitalizations (17%) in the form of UTI, pneumonia, osteomyelitis, and fever.

Acute chest syndrome emerged as the third leading reason for hospitalization (11%), followed by acute hemolytic crisis (9%) (Table 2). In our patient population, 60%, 21%, and 12% were presented with joint pain, back pain, and chest pain at the time of admission (Table 3).

**Table 2:** Relation between the causes of hospitalization and age group among the study group Notes: n = number, chi-square test; *Significant value

Cause of Hospitalization	Age Group (n=100)		P Value
	>15-19(n= 25)	>19 (n = 75)	
Painful crisis (n=63)	17 (27%)	46 (73%)	<0.000*
Infection (n=17)	5 (29.41%)	12 (66.66%)	0.09
Acute chest syndrome (n=11)	1 (9.09%)	10 (90.90%)	0.007*
Acute hemolytic crisis (n=9)	2 (22.22%)	7 (77.77%)	0.096

**Table 3:** Relation between pain and age group among members of the study group Notes: n = number, chi-square test; *Significant value

Pain Symptoms at the Time of Admission	Age Group (n=100)		P Value
	>15£19 (n=25)	>19 (n=75)	
Joint pain (n=60)	19 (31.66%)	41 (68.33%)	0.005*
Back pain (n=21)	3 (14.28%)	18 (85.71%)	0.001*
Chest pain (n=12)	1 (8.33%)	11 (91.66%)	0.001*
Abdominal pain (n=7)	2 (28.57%)	5 (71.42%)	0.257

Upon examining the reasons for hospitalizations in different age groups, a notably greater proportion of individuals experienced painful crises (VOC) (p = < 0.000) and acute chest syndrome (p = 0.007), and adults>19 years old more commonly presented the symptoms of joint pain (p = 0.005), back pain (p = 0.001), and chest pain (p = 0.001) (Table 2). In terms of the association between the underlying reasons for hospitalizations and gender, our investigation revealed no significant relation between males and females concerning the reasons for hospitalizations (p > 0.05).

As shown in Table 4, the incidence of acute painful crisis in cases having hemoglobin F (HbF) ≤20% was 66.66% (n = 62), while that in cases having HbF ≥20% was 33.33% (n = 32). The incidence of vaso-occlusive crisis in cases having HbF ≤20% was 65.75% (n = 48), while that in cases having HbF ≥20% was 34.24% (n = 25). The incidence of acute chest syndrome in cases having HbF ≤20% was 71.79% (n = 28), while that in cases having HbF ≥20% was 28.2% (n = 11). The incidence of acute hemolytic crises in cases having HbF ≤20% was 73.13% (n = 49), while that in cases having HbF ≥20% was 26.86% (n = 18). The incidence of fever in cases having HbF ≤20% was 65.45% (n=36), while that in cases having HbF ≥20% was 34.54% (n = 19). In terms of the association between the complications and HbF value, the results were statistically significant (p = 0.0056).

**Table 4:** Relation between HbF percentage and acute clinical events among members of the study group HbF, Hemoglobin F

Acute Clinical Events	HbF Percentage		
	<20% (cases=62)	>20% (cases=38)	Total (cases=100)
Vaso-occlusive crisis	48	25	73
Acute chest syndrome	28	11	39
Avascular necrosis	5	2	7
Abdominal pain	24	21	45
Fever	36	19	55
Leg ulcer	5	0	5
Splenomegaly	18	17	35
Hepatomegaly	22	12	34
Urinary tract infection	2	2	4
Osteomyelitis	3	2	5
Thrombocytopenia	1	3	4
Pneumonia	4	1	5
Hepatitis	2	0	2
Stroke	2	2	4
Acute hemolytic crisis	49	18	67
Chronic liver disease	4	5	9
Peptic ulcer disease	2	1	3
Acute painful crisis	64	32	96
Heart failure	2	0	2

**Figure 2:**
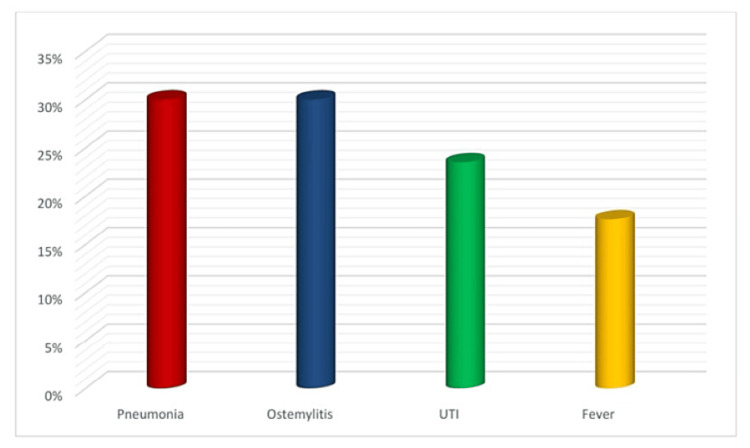
Bar chart showing the percentage of different types of infections among SCD patients SCD, Sickle cell disease

Patients who had an infection had a considerably longer hospital stay compared to those who experienced painful crises in our study population (p = 0.040). However, there was no statistically significant difference in the duration of hospital stay between age and gender (Table 5). Pneumonia and osteomyelitis (30%) constitute greater proportions of infections in our study group (Figure 2).

**Table 5:** Duration of hospital stay of the selected variables among patients with SCD 𝑃 value calculated by ANOVA for causes and by 𝑡-test for other variables. ^∗^The significant difference between infection and painful crises in relation to the duration of stay.

Variable	Duration of Hospital Stay (Mean ± SD)	P Value
Age		
>15-19	8.84 ± 6.01	0.352
>19	10.33 ± 7.17	
Sex		
Male	10.67 ± 7.14	0.214
Female	8.92 ± 6.48	
Causes		
Painful crisis	10.59 ± 7.08	0.04*
Infection	12.68 ± 8.7	
ACS	7.27 ± 1.42	
AHC	5.77 ± 1.20	

## Discussion

Any intervention aimed at preventing hospital admission in SCD may reduce the complications and economic burden of the disease [19]. This study evaluates the reasons for hospitalization among adolescent and adult patients with SCD, and it reveals that painful crises or VOC, infections, acute chest syndrome (ACS), and hemolytic crisis are the primary causes of hospital admissions in descending order of frequency.

VOC or painful crises, caused by the hindrance of microcirculation by sickled RBCs, is a significant complication of SCD, resulting in ischemic injury of the affected organ and pain. VOC is the most distinct clinical feature of SCD, accounting for most emergency department visits and hospitalizations in affected patients. This study investigated hospitalization causes among adolescents and adults with SCD and found that VOC was the leading cause, accounting for 63% of admissions. These findings align with previous studies conducted in Saudi Arabia and Iraq, which reported VOC as the primary cause of hospitalizations in 91.3% and 73.8% of admitted patients, respectively [11,12]. In numerous studies, VOC has been identified as a prevalent factor leading to hospital admissions. This may be linked to the fact that these patients engage in physical activity without adequately maintaining proper hydration, resulting in susceptibility to dehydration and thrombotic events [20]. Given that a painful crisis or VOC is the primary cause of hospitalization in individuals with SCD, it is essential to provide them with a comprehensive education on the factors that trigger such crises. Specifically, patients must be advised to avoid excessive physical exertion and exposure to extreme weather conditions, as well as the importance of maintaining adequate hydration levels [21].

SCD is characterized by recurrent episodes of severe pain that necessitate hospitalization. Personalized pain management in the emergency department has been shown to enhance the quality of care for such crises, leading to high levels of patient satisfaction and reduced rates of avoidable hospitalizations [8]. In our study, the majority of SCD patients exhibited occurrences of joint pain, back pain, and chest pain with a noticeably greater prevalence among those aged above 19 years. Bone pain during VOC is attributable to bone marrow infarction, which primarily involves the bones where the marrow is actively engaged, and this varies based on the age of the patient.

In the present study, infection emerged as the second leading reason for hospital admissions (17%) among SCD patients. This finding is like a study conducted in Basra, Iraq, where infections were identified as the second most frequent cause of hospitalization, accounting for 9.3% of all cases [11]. Patients diagnosed with SCD are susceptible to severe bacterial infections due to various factors, but poor splenic function is the most significant factor [17]. ACS is responsible for causing most of the morbidity and mortality in both pediatric and adult populations with SCD, comprising approximately 25% of deaths. This condition can arise after VOC due to hypoxia induced by chest hypoventilation.

Additionally, ACS can manifest because of fat embolism originating from the distal bone during a crisis. Among our patients, ACS was the third most common cause of hospitalization (11%). This finding is similar to an earlier study conducted by Salman et al., who identified ACS as the third most common reason for admission among patients [11]. In our study, hospitalization due to hemolytic crisis was identified as the least common cause (9%) with a mean hemoglobin (Hb) concentration of 8.05 ± 1.85 g/dL. This finding is the same as in a study conducted by Elmoneim et al. with a mean Hb concentration (8.1 ± 2.1 g/dL) [22].

In this study, we examined the relationship among various reasons for hospitalizations and patients' age and gender. Our analysis revealed that there was no significant association between gender and the different reasons for hospitalizations among adolescent and adult patients with SCD. When we looked at the reasons for hospitalizations about age group, we found that VOC with joint, back, and chest pains were more prevalent among adult patients with SCD. The findings of this study indicate that the length of hospitalization for patients with SCD did not show any significant difference based on age or gender. The mean duration of hospital stay for patients with infection was longer than that of patients with painful crises. However, our study does not agree with what has been described earlier that the mean duration was longer for patients with acute chest syndrome than for patients with acute splenic sequestration crises [11].

The limitations of the study are that it is a single-center study catering to a particular geographic location in India, the sample size is small, and the follow-up of the patients is lacking.

## Conclusions

Based on the findings of this research, it can be inferred that, even though acute painful crises were the primary reason for hospitalization in individuals with SCD, a significant number of patients also experienced infections and acute chest syndrome. Moreover, patients suffering from various infections and VOC had a longer hospital stay. It is thus of prime importance to provide them with comprehensive education on the factors that trigger such crises. Patients must be advised to avoid excessive physical exertion and exposure to extreme weather conditions and should be explained the importance of maintaining adequate hydration levels, especially in warm weather.
